# FoxG1 as a Potential Therapeutic Target for Alzheimer’s Disease: Modulating NLRP3 Inflammasome via AMPK/mTOR Autophagy Pathway

**DOI:** 10.1007/s10571-024-01467-4

**Published:** 2024-04-17

**Authors:** Qi Yun, Si-Fei Ma, Wei-Ning Zhang, Meng Gu, Jia Wang

**Affiliations:** 1 Changzhou Children’s Hospital Affiliated to Nantong University, 958 Zhongwu Avenue, Changzhou, 213000 Jiangsu Province China; 2 Changzhou Blood Center, 118 Canal Road, Changzhou, 213000 Jiangsu Province China; 3 Department of Laboratory Medicine, School of Medicine, Jiangsu University, 301 Xuefu Road, Zhenjiang, 213000 Jiangsu Province China; 4 The Fourth Affiliated Hospital of Jiangsu University, Zhenjiang, 212001 Jiangsu Province PR China

**Keywords:** FoxG1, Alzheimer’s disease, β-amyloid, NLRP3, Autophagy, AMPK/mTOR

## Abstract

An increasing body of research suggests that promoting microglial autophagy hinders the neuroinflammation initiated though the NLRP3 inflammasome activation in Alzheimer’s disease (AD). The function of FoxG1, a crucial transcription factor involved in cell survival by regulating mitochondrial function, remains unknown during the AD process and neuroinflammation occurs. In the present study, we firstly found that Aβ peptides induced AD-like neuroinflammation upregulation and downregulated the level of autophagy. Following low-dose Aβ25–35 stimulation, FoxG1 expression and autophagy exhibited a gradual increase. Nevertheless, with high-concentration Aβ25–35 treatment, progressive decrease in FoxG1 expression and autophagy levels as the concentration of Aβ25–35 escalated. In addition, FoxG1 has a positive effect on cell viability and autophagy in the nervous system. In parallel with the Aβ25–35 stimulation, we employed siRNA to decrease the expression of FoxG1 in N2A cells. A substantial reduction in autophagy level (Beclin1, LC3II, SQSTM1/P62) and a notable growth in inflammatory response (NLRP3, TNF-α, and IL-6) were observed. In addition, we found FoxG1 overexpression owned the effect on the activation of AMPK/mTOR autophagy pathway and siRNA-FoxG1 successfully abolished this effect. Lastly, FoxG1 suppressed the NLRP3 inflammasome and enhanced the cognitive function in AD-like mouse model induced by Aβ25–35. Confirmed by cellular and animal experiments, FoxG1 suppressed NLRP3-mediated neuroinflammation, which was strongly linked to autophagy regulated by AMPK/mTOR. Taken together, FoxG1 may be a critical node in the pathologic progression of AD and has the potential to serve as therapeutic target.

**Supplementary Information:**

The online version contains supplementary material available at 10.1007/s10571-024-01467-4.

## Introduction

Alzheimer’s disease (AD) is a neurodegenerative disorder that progresses over time, leading to the gradual decline of cognition and memory (Martínez et al. 2012; Hradek et al. 2015). The AD pathologies are frequently linked to significant neuronal harm and the existence of senile plaques, especially concentrated in the brain regions where neurodegeneration takes place (Wei et al. 2019). The main component of these plaques associated with old age is the amyloid-peptide (Aβ), which is formed from amyloid precursor protein (APP) via a series of cleavages by the β-secretase and γ-secretase complex (Li et al. 2020; Qiu et al. 2020). Research has indicated that the majority of AD cases are sporadic, occurring due to the hindered clearance of Aβ (Tao et al. 2022). Previous studies indicates that the increase in neuroinflammation caused by Aβ is the mechanism through which this peptide exerts its neurotoxic effects in both in AD (Qiu et al. 2020; Hong et al. 2019). Inflammation may be a key enabler of AD pathological processes, which may shatter the balance between cell survival and cell death in AD patients’ pathology (Zhong et al. 2017). The NLRP3 inflammasome, which is the predominant inflammasome, is widely expressed in microglia and can be triggered by different viral. The NLRP3 inflammasome plays a crucial part in the regulation of neuroinflammation and the pathological progression of AD(Van Zeller et al. 2021). According to research, concomitantly activation of NLRP3 inflammasome, numerous pro-inflammatory cytokines generated, like IL-6 and TNF-α, ae well as GFAP (Ren et al. 2019; Babić Leko et al. 2020), more specific in the nervous system (Hol and Pekny 2015), leading to an increased rate of neuronal death and the degradation of AD. The activation of NLRP3 inflammasome may lead to a decline in phagocytic function in AD (Nizami et al. 2019). Individuals with AD suffer from an excessive inflammatory response in their brain’s microglia, which reduces the autophagy level or impairs the autophagy pathway as compared to those who are healthy (Zhou et al. 2020; Wen et al. 2019). The regulation of neuroinflammation and reduction of Aβ deposition through autophagy are an essential and widely conserved process in all eukaryotic cell (Parzych and Klionsky 2014). Maintaining cellular protein homeostasis requires this essential process of cellular recycling, which supplies the cell with raw materials by systematically breaking down senescent and dysfunctional organelles (Kim and Lee 2014). The deficiency in the level of autophagy plays a significant role in the development of AD, which involves the gradual accumulation of Aβ and the hindrance of Aβ breakdown (Reddy and Oliver 2019). 3-methyladenine (3-MA), acting as an inhibitor of autophagy, can effectively be utilized to inhibit mitochondrial autophagy (Shi et al. 2020). In contrast, rapamycin (Rap), a widely used autophagy inducer, can suppress the inflammatory response by reducing the expression of NLRP3 inflammasome. This helps maintain the proper functioning of microglia and improve the survival and activity of neurons (Boada et al. 2020; Zhang et al. 2021). Therefore, it is believed that enhancing autophagy to suppressing the NLRP3 inflammasome could be a beneficial approach for treating AD.

FoxG1, is a winged-helix gene family member encoded by the FOXG1 gene located at 14q12 (Obendorf et al. 2007; Yao et al. 2001). FoxG1 chiefly localizes in the nucleus and is commonly restricted to the nervous system (Seoane et al. 2004). The research findings indicated that FoxG1 is typically present in forebrain progenitor cells from development to adulthood (Fasano et al. 2009). FoxG1, as a transcription factor, plays a crucial role to regulate cellular growth and development (Adesina et al. 2015). Nevertheless, alterations in the FOXG1 gene impact the growth of neurons and can potentially trigger a profound neurodevelopmental condition referred to as FOXG1 syndrome (previously recognized as a cognitive variation of Rett syndrome) (Guerrini and Parrini 2012). And patients suffer from epileptic seizure, mental retardation, and abnormal social behaviors (Zhang et al. 2017). Therefore, aberrant expression of FoxG1 will make negative effects for neuron development. However, FoxG1 is rarely reported in neurodegenerative diseases.

Autophagy modulation is mediated by kinases, including mammalian target of rapamycin (mTOR) and AMP-activated protein kinase (AMPK) (Li et al. 2019). AMPK, which acts as an energy detector, governs cellular metabolism and balance, facilitating autophagy. Conversely, mTOR, a serine/threonine protein kinase, acts as a suppressor of autophagy that governs cell growth, proliferation, movement, and survival (Chun and Kim 2021; Assaf et al. 2022). It can be seen that AMPK/mTOR pathway plays a decisive role in autophagy process. Furthermore, changes in numerous proteins can suggest that autophagy occurs, including the autophagy inducer Beclin1 (Beclin1), microtuble-associated protein 1 light chain 3 (LC3), and autophagy adaptor p62 (SQSTM1/P62). It is known that autophagy activation may have an anti-inflammatory effect in AD (Freitag et al. 2022). Nevertheless, the functions of FoxG1 in the regulation of autophagy and its association with AD pathology has yet to be clarified. To investigate the role of FoxG1 in AD, we construct AD cell model induced by the Aβ_25–35_ drug and APPswe plasmid (Rao et al. 2023; Dai et al. 2021; Gao et al. 2020). Our study aimed to uncover the mechanisms and impacts of FoxG1 in protecting against inflammation-induced autophagy level degeneration in AD. During the administration of Aβ_25–35_ drug at low concentrations, we observed a gradual increase in the expression of FoxG1 and autophagy level. However, with high dose of Aβ_25–35_ drug, there was a sharp decline in both FoxG1 expression and autophagy level. In addition, we investigated the correlation between FoxG1 and the process of autophagy, revealing that FoxG1 controls the AMPK/mTOR autophagy pathway and sustains intracellular homeostasis. And here, by crossing *Cre*^*ER*^ with *FoxG1*^*fl/fl*^ lines combined with tamoxifen (TM) induction, and further injecting Aβ_25–35_ drug into the lateral ventricle, FoxG1 was conditionally overexpressed in mature neurons in Aβ_25–35_-induced AD *vivo* model and to continue exploring the effects of FoxG1 in improving pathology of AD. In a word, FoxG1 could potentially serve as the crucial protein enhance autophagy by the AMPK/mTOR pathway to suppress NLRP3 inflammasome in AD.

## Materials and Methods

### Chemicals and Reagents

The Aβ_25–35_ (Sigma, St. Louis, MO, USA) is the shortest fragment of Aβ that is processed in vivo by brain proteases. This peptide retains the ability to self-aggregate and elicits essentially the same toxicity as the full-length peptide despite lacking the hydrophobic C terminal sequence of five amino acids that is present in Aβ_1–40_. The Aβ_25–35_ was respectively aggregated by incubation in distilled water for 1 mg/mL and 500 μM at 37 °C for 7 days before the *vivo* and *vitro* experiment, and the solution is eventually stored at minus 20 degrees Celsius. Tamoxifen (TM, T5648) and corn oil (C8267) were purchased from Sigma (St. Louis, MO, USA). 3-methyladenine (3-MA, HY-19312) and rapamycin (Rap, HY-19312) were obtained from MCE Co., Ltd. (Shanghai, China). Puromycin (A1113803) was purchased from Thermo Fisher (Massachusetts, USA).

### Antibodies and Plasmids

The primary antibodies used in this study include rabbit anti-FoxG1 (ab18259) and mouse anti-SQSTM1/P62 (ab56416) antibodies from Abcam (Cambridge, UK); rabbit anti-LC3A/B (12,741), rabbit anti-p-mTOR (5536), rabbit anti-p-AMPK (2535), and mouse anti-Aβ (15,126) antibodies from Cell Signaling Technologies Inc. (MA, USA); rabbit anti-AMPK (WL02254), rabbit anti-mTOR (WL02477), rabbit anti-BAX (WL01637), and rabbit anti-Bcl2 (WL01556) antibodies from Wanleibio (ShengYang, China); rabbit anti-GFAP (bs-0199R) antibodies from Bioss Biotechnology Co. (Beijing, China); rabbit anti-Beclin1(A7353) and rabbit anti-NLRP3(A5652) were purchased from ABclonal Biotechnology Co. (Wuhan, China); and mouse anti-β-tubulin (AF2835), mouse anti-GAPDH (AF2819), and mouse anti-GFP (AF0159) from Beyotime Biotechnology (Shanghai, China). The plasmids, including pEGFP in PCAX APPswe Ind (C096) was purchased ABM (Vancouver, Canada); pECMV-Foxg1-m-FLAG (P6422) and pCDH-CMV-MCS-EF1-CopGFP-T_2_A-Puro (P13480), psPAX2 (P0261) and pMD2.G (P0262) were purchased from Miaolingbio (WuHan, China).

### Plasmid Construction

A pECMV-Foxg1-m-FLAG sequence was synthesized commercially and constructed in pCDH vector to establish the pCDH-FoxG1-m-FLAG-CopGFP-T_2_A-Puro expressing plasmid according to the manufacturer’s instructions.

### Cell Culture and Generation of Stable Cell Lines

N_2_A cells and HEK293 cells were purchased from American Type Culture Collection (ATCC; Rockville, MD, USA). They were cultured in Dulbecco’s modified Eagle’s medium (DMEM, BI, Israel) supplemented with 10% Fetal bovine serum (BI, Israel), 50 U/Ml penicillin, and 50 μg/mL streptomycin. All cells were maintained in a 5% humidified CO_2_ incubator at 37 °C. To generate cell populations stably N_2_A cells expressing FoxG1-FLAG (FoxG1/N_2_A) and pCDH vector (pCDH/N_2_A), the control pCDH or the pCDH-FoxG1-FLAG, psPAX2, and pMD2.G plasmids were transfected into HEK293 cells with Lentifectin™ Transfection Reagent (G074, ABM, Vancouver, Canada) according to the manufacturer’s instructions. After 48 h of transfection, stable transfectants were selected in medium containing 1 μg/mL puromycin for 7–14 days. After two or three passages in the presence of puromycin, the cultures were used for experiments. GFP-positive clonings were detected by fluorescence microscope (Olympus, Japan).

### Animals

All mice were housed in a room (humidity, 60%; temperature, 20–24 °C) under a 12 h light/dark cycle. All mice had free access to food and water ad libitum at the Jiangsu University. All experimental procedures followed the guidelines approved by Jiangsu University. CAG-loxp-stop-loxp-FoxG1-IRES-EGFP (*FoxG1*^*fl/fl*^) mice were kindly provided by Dr. Chunjie Zhao (Medical School of Southeast University, China). Genotypes of the offspring were determined by PCR analysis using primers for (5′-AAG GAC GAC GGC AAC TAC AAG-3′ and 5′-GGC GGT CAC GAA CTC CA-3′) to amplify a 378 bp FoxG1 fragment. B6;129–5-HT1B-tm1(*Cre*^*ERT*^) mice were purchased from Model animal research center of Nanjing University (T000414, Nanjing, China). Genotypes of the offspring were determined by PCR analysis using primers for (5′-AAGCTAAGTTCCTCGGGTATGGAAG-3′, 5′-TCT GAG GCG GAA AGA ACC AG-3′ and 5′-CTT CTT CAT CAT CTC CCT GGT GAT G-3′, 5′-AAG CTA AGT TCC TCG GGT ATG GAA G-3′). To overexpress FoxG1 in the hippocampus, the *FoxG1*^*fl/fl*^ males were crossed with *Cre*^*ERT*^ females to achieve *Cre/FoxG1*^*fl/fl*^ double transgenic mice, and TM was administered by intraperitoneal injection to *Cre/FoxG1*^*fl/fl*^ mice. TM was dissolved in corn oil at a concentration of 20 mg/mL, and the dose administered was 75 mg/kg body weight once a day and sacrificed at day 14 to detect the changes in FoxG1 expression in hippocampus (Wang et al. 2022a, c).

### Development of AD Mouse Model and Drug Treatments

*Cre/FoxG1*^*fl/fl*^ mice were anesthetized with 50 mg/kg sodium pentobarbital (i.p. P11011, Sigma-Aldrich, USA) and placed in a stereotaxic instrument (RWD, Shenzhen, China). The aggregated Aβ_25–35_ was injected into the lateral ventricle with the following coordinates: 2.0 mm posterior to bregma, 2.0 mm lateral to the midline, and 1.7 mm beneath the surface of the brain. Sham-operated mice were injected in the same manner with sterile saline. Five microliters of aggregated Aβ (500 μM) was injected into the right lateral ventricle using a stepper-motorized micro-syringe at a rate of 0.3 μl/min. The syringe was removed after completing the injection 10 min. Twenty-four hours later, *Cre/FoxG1*^*fl/fl*^ mice were divided into five groups: the sham-operated control group, Aβ_25–35_-intoxicated group, TM-treated group, TM and Aβ_25–35_-intoxicated group (7 mice in each group). Seven days before starting the injection Aβ_25–35_, TM and Aβ_25–35_-intoxicated group mice were intraperitoneal injection TM once a day for consecutive 14 days.

### Morris Water Maze Test (MWM)

Spatial learning ability was tested in the MWM test as our previous report (Wang et al. 2020a). The acquisition phase is performed from day 1 to day 6, and on day 7, a probe test is performed in which the escape platform is removed. The reversal learning phase is conducted from day 8 to day 10, followed by a second probe test 24 h later (day 11). Escape latency and path length to reach the hidden platform during the acquisition and reversal phase for animals are recorded. Duration spent in the target zone of the water maze pool during probe tests are tracked and digitized, and the information is stored for subsequent behavioral analysis.

### Novel Object/Place Learning

The novel object/place recognition task was tested in 3 phases: habituation, sample, and test, as described in our previous report (Wang et al. 2020a). The duration (time) of explorations of each object/place was recorded and expressed as a discrimination index for the test phase. The index was associated with the duration of exploration (D) of the novel object/place (DN) and the familiar object/place (DF) and total duration of exploration of the novel and familiar objects [Index = (DN − DF)/(DN + DF)]. Between sessions and animals, the arena and objects were cleaned with 70% ethanol to eliminate olfactory cues.

### Flow Cytometry

The apoptosis rate of N_2_A cells was measured by flow cytometry using the Annexin V staining kit (KeyGEN BioTECH, NanJing, China) according to the manufacturer’s instructions. Briefly, N_2_A cells were collected and centrifuged at 800 rpm for 5 min as the treatments were finished. The supernatant was then removed, and the cell pellet was gently re-suspended with 2000 μL 1 × Annexin V solution containing 5 μL FITC and 5 μL propidium iodide (PI) staining reagents. After an incubation for 5–15 min in dark, N_2_A cells were analyzed by the BD FACSVerse Flow Cytometer System (BD Biosciences, San Jose, CA, USA) according to the manufacturer’s instructions. Data acquisition and analysis were performed using FlowJo software (ACEA Biosciences).

#### Immunofluorescence analysis

After treatment, N_2_A cells were fixed in 4% PFA solution for 30 min at room in dark, followed by washing with PBS 3 times and a permeabilization with 0.1% Triton X-100 for 30 min. Then, coverslips were blocked with 10% goat serum for 1 h and then incubated with primary antibodies (1:50) overnight at 4 °C. After incubation, the cells were washed with PBS 3 times and further incubated with Alexa Fluor 488 anti-mouse secondary antibody (A-32723, Invitrogen Massachusetts, USA) or Alexa Fluor 594 anti-rabbit secondary antibody (A-11037, Invitrogen, Massachusetts, USA) (1:500) for 1 h, and then stained with DAPI for 5 min at room temperature; the cells were washed with PBS 3 times. Next, the coverslips were detected under fluorescence microscope. The representative images were captured by fluorescence microscope (Olympus, Japan).

#### Immunohistochemistry

Mouse brains in 4% PFA were dehydrated in 30% sucrose and frozen in optimal cutting temperature (OCT) compound embedding medium. The frozen sections were cut into 12 μm slices containing the hippocampus and fixed on glass slides. After blockage with 10% goat serum, the slices were incubated with rabbit anti-FoxG1 antibodies at 4 °C overnight, followed by incubation with HRP-conjugated goat anti-mouse secondary antibody (diluted 1:300) at room temperature for 90 min. After washing, DAB (SV0004, Bosterbio, Wuhan, China) was used for dyeing, and hematoxylin was used for retaining. The representative images were captured by an optical microscope (Olympus, Japan), and the optical densities representing the protein expressions in the hippocampus of brain sections were analyzed by Image J software (National Institutes of Health, Bethesda, MD, USA).

#### Transfection

N_2_A cells seeded on the coverslips in 6-well plates were transfected with pEGFP-APPswe, pECMV-Foxg1 plasmids, and siRNA-FoxG1 1–3; siRNA control RNA is using the Lipofection 2000 (11668019, Thermo Fisher, Massachusetts, USA) according to the manufacturer’s instructions. After transfection for 18–24 h, N_2_A cells were treated with the indicated drugs for 24 h. Then, the cells proceeded to the next corresponding other processing. The sequences for siRNA-FoxG1 1–3 (m-FoxG1-1141, m-FoxG1-1509 and m-FoxG1-1990) and control siRNA (Sangon Biotech, Shanghai, China) were listed as follows: sense 5′-CCU GAC GCU CAA UGG CAU CUA TT-3′, antisense 5′-UAG AUG CCA UUG AGC GUC AGG TT-3′ and sense 5′-GCA CUU UGA GUU ACA ACG GGA TT-3′, antisense 5′-UCC CGU UGU AAC UCA AAG UGC TT-3′ and sense 5′-UUC CAA CCC UUU AAU ACA UUA TT-3′, antisense 5′-UAA UGU AUU AAA GGG UUG GAA TT-3′ and sense 5′-UUC UCC GAA CGU GUC ACG UTT-3′, and antisense 5′-ACG UGA CAC GUU CGG AGA ATT-3′.

#### Western Blot

After treatment, N_2_A cells and hippocampus were lysed with cold RIPA Lysis Buffer plus PMSF. The lysates were collected for centrifugation at 14,000 rcf for 10 min at 4 °C, the supernatant was then transferred into 1.5 mL tubes, and the protein concentration was measured on the basis of the manufacturer’s instructions. The same amount of protein is loaded in each sample onto SDS-PAGE for electrophoresis. Next, the proteins on the gel were transferred onto the poly-vinylidene fluoride (PVDF) membrane (Millipore, Billerica, MA). The membrane was then blocked with 5% BSA in TBST and incubated with the primary antibodies overnight at 4 °C. After incubation, the membrane was washed with TBST 3 times and incubated with the HRP-conjugated secondary antibodies for 60 min at room temperature. After incubation and washing with TBST 5 times, the bands on the membrane were revealed by the ECL detection reagent (MA0186, Meilunbio, ShangHai, China). The western blots were semi-quantified using Image J to measure the intensities of the bands.

#### Real-Time PCR

Total RNA was extracted from N_2_A cells with RNAiso Plus (108-95-2, TAKARA, Kusatsu, RShiga) and reverse transcribed to cDNA by using PrimeScript™ RT reagent Kit with gDNA Eraser (RR047A, TAKARA, Kusatsu, Shiga) according to the manufacturers’ protocols. The RT-PCR was performed on a CFX96 Rt-PCR system (Bio-Rad, USA) with the UltraSYBR Mixture (CW0957, CWBiotech, Beijing, China). The following primers were designed for each targeted mRNA: FoxG1, sense 5′- TCA TCA TGA TGG CCA TCC GG-3′ and antisense 5′- GCA CCT TCA CGA AGC ACT TG-3′; GAPDH, sense 5′- GAG AAG GCT GGG GCT CAT TT-3′ and antisense 5′- GAG AAG GCT GGG GCT CAT TT-3′; AMPK, sense 5′-CCC ATG AAG AGG GCC ACA AT-3′ and antisense 5′-GCC CTT GGT GTT TCA GCA AC-3′; mTOR, sense 5′-GTG CCG AGC ATA TGC CAA AG-3′ and antisense 5′-GAC CTA AAC CCC ATG CAG CT-3′. RT-PCR conditions consisted of an initial denaturing step of 10 min at 95 °C followed by 40 cycles of 10 s denaturation at 95 °C, 30 s annealing at 58 °C, and 32 s extension at 72 °C. The mRNA expression was normalized to the mRNA expression of GAPDH. The results were calculated using the comparative cycle threshold (ΔΔCt) method.

#### Electron Microscopy

The mouse brains were fixed in 2.5% glutaraldehyde (P1126, Solarbio, BeiJing, China) for 24 h, immobilized in 1% osmic acid (SigmaAldrich, O5500) for 1–2 h, dehydrated with acetone (Sinopharm Chemical Reagent, u1006801), and embedded in araldite CY 212(TAAB, E009). Ultrathin sections were stained with alcoholic uranyl acetate (Polysciences, 6159-44-0) and alkaline lead citrate (SigmaAldrich, 15326). The sections were washed gently with distilled water and observed with a JEM 1230 TEM (JEOL Ltd., Tokyo, Japan).

#### Cell Counting Kit-8 (CCK8) assay

Cell viability assays were performed using the Cell Counting Kit-8 (BA00208, Bioss, BeiJing, China). Cells were seeded with culture medium onto 96-well plates (1 × 105 cells/mL; 100 μL) and incubated at 37 °C for 24 h. After adaptation, cells were treated in combination for 24 h. Then, the culture medium was replaced with fresh medium containing 10 μL of CCK-8 solution. The optical density (OD) at 490 nm was assayed after cell incubation at 37 °C for 2 h.

#### Imaging for Autophagic Flux

The HBAD-mCherry-EGFP-LC3 adenoviral particles were purchased from HanBio (Shanghai, China). Cells were infected with adenoviral particles. After treatment, N_2_A cells were washed with PBS and fixed with 4% PFA for 20 min at room temperature. The slides were air dried and detected autophagy flux using the Laser scanning confocal microscope (Olympus, Japan) with 60× magnification. Representative images of GFP-LC3 and RFPZ-LC3 were captured, and then the EGFP and RFP fluorescence images with the same field were merged to detect the autophagy flux by calculating the EGFP/RFP ratio using the ImageJ software.

#### Statistical Analysis

All data in this study were obtained from at least three independent experiments. Data expressed as the mean ± SEM (standard error of mean) were analyzed by the StatView 5.01 software using one-way ANOVA followed by Fisher’s protected least significant difference test. A *p* value < 0.05 was considered to have statistical significance among the groups.

## Results

### FoxG1 Induces Autophagy and Decreases Cell Death Caused by Autophagy Inhibited in N_2_A Cells

**Fig. 1:**
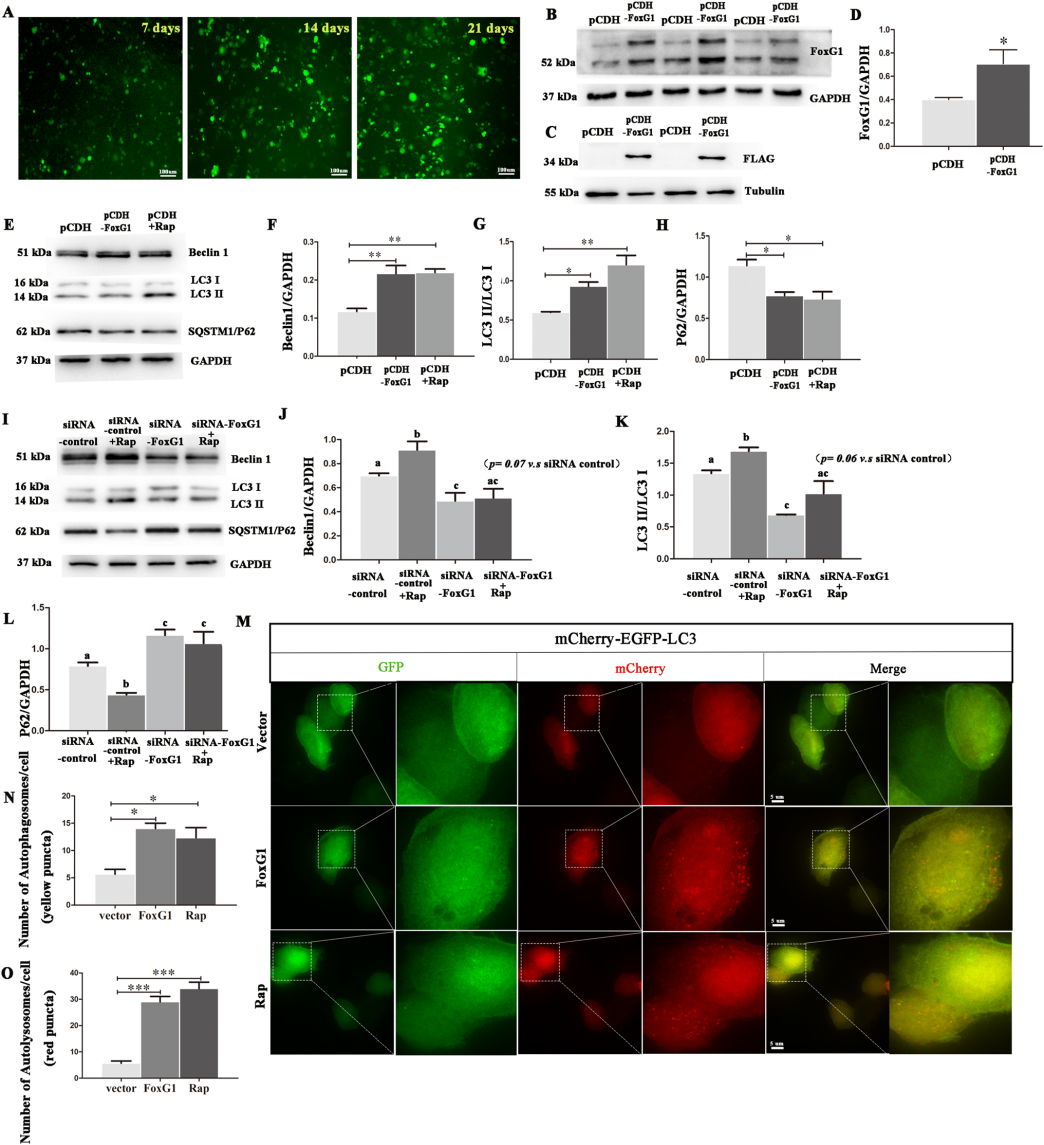
Construction of FoxG1 stable expression cell lines and FoxG1 induces autophagy in N_2_A cells. Stable expression of pCDH-FoxG1-m-FLAG-CopGFP-T_2_A-Puro in mouse N_2_A cell lines. N_2_A was stably transfected with pCDH-FoxG1-m-FLAG-CopGFP-T_2_A-Puro or pCDH-CopGFP-T_2_A-Puro and selected with puromycin-containing medium. Cell lines were named as FoxG1/N_2_A and pCDH/N_2_A cells, respectively. **A** Green fluorescent proteins are screened positive clones after using 1 μg/mL puromycin 7 days, 14 days, and 21 days, respectively. Scale bar = 100 μm. Expression patterns and qualification of FoxG1 expression in the FoxG1/N_2_A cell lines and pCDH/N2A cell lines were studied by (**B**, **D** [*t*_11_ = 3.496, *P* = 0.025]) western blotting across the protein bands. **C** Anti-FLAG antibody was also used to check the successful construction of FoxG1 stably expressing cell lines after 14–21 days of puromycin screening. GAPDH or Tubulin was used as the relative loading control. **E** pCDH/N_2_A cells were treated with 20 nM Rap for 24 h, protein was then harvested for the detection of Beclin1 and LC3 and SQSTM1/P62 by Western blot in pCDH/N2A cells and FoxG1/N2A cells and Rap treating pCDH/N2A cells. (**G** [*F*_2,15_ = 15.92, *P* = 0.004]) Bar chart indicates the relative expression of LC3 II/LC3 I in (**E**). (**F** [*F*_2,15_ = 16.03, *P* = 0.0039], **H** [*F*_2,15_ = 10.32, *P* = 0.0114]) Bar chart indicates the relative expression of these proteins in E. GAPDH was used as loading control. **I** N_2_A cells transiently transfected with siRNA controller siRNA-FoxG1 for 24 h were treated with 20 nM Rap for 24 h. After treatment, cells were harvested for the detection of Beclin1 and LC3 and SQSTM1/P62 by Western blot. (**K** [*F*_3,20_ = 16.71, *P* = 0.0008]). Bar chart indicates the expression of LC3 II/LC3 I in (**I**). (**J** [*F*_3,20_ = 9.676, *P* = 0.0049], **L** [*F*_3,20_ = 15.44, *P* = 0.0011]) Bar chart indicates the relative expression of these proteins in I. GAPDH was used as loading control. **M** Cells were infected with mcherry-GFP-LC3 adenovirus and transfected with vector or FoxG1 plasmid or treated with Rap. Yellow dots indicate autophagosomes and red dots indicate autolysosomes. (**N** [*F*_2,12_ = 11.17, *P* = 0.0095]) Quantification of autophagosomes in (**M**). (**O** [*F*_2,12_ = 59.95, *P* = 0.0001]) Quantification of autolysosomes in (**M**). Scale bar = 5 μm. For all experiments, different letters indicate statistical differences in mean values across groups (*P* < 0.05). Values are expressed as means ± SEM. *P < 0.05, **P < 0.01,***P < 0.001. For each group, *n* = 6/group

In this study, to explore the relationship between FoxG1 and autophagy in N_2_A cells, we make a thorough inquiry the expression of LC3II/LC3I, autophagy inducer Beclin1 and the degradation of the autophagic substrate SQSTM1/p62 protein when the expression of FoxG1 changes in N_2_A cells. First, to increase the expression of FoxG1, we constructed N_2_A cell lines stably expressing pCDH-FoxG1-m-FLAG-CopGFP-T_2_A-Puro (FoxG1/N_2_A cell lines) and negative control pCDH-CopGFP-T_2_A-Puro (pCDH/N_2_A cell lines) for all of the experiments in this study. Compared with negative control, we confirmed the overexpression of FoxG1 using anti-FoxG1 and anti-FLAG antibodies by western blot after 14–21 days of puromycin screening (Fig. 1A–D, *P* < 0.05). Meanwhile, the western blot data showed that the expressions of LC3 II/LC3 I ratio and Beclin1 were significantly increased and SQSTM1/p62 was decreased in FoxG1/ N_2_A cell lines compared with pCDH/N_2_A cell lines, and the autophagy activator rapamycin (Rap) was acted as positive control (Fig. 1E–H, *P* < 0.05). Next, we used siRNA transfection to knock down FoxG1 expression in N_2_A cells. We found that downregulation of FoxG1 could reduce LC3 II and Beclin1 proteins expression and increase the expression of SQSTM1/P62 protein (Fig. 1I–L, *P* < 0.05). Further, the Rap-induced changes in LC3II/LC3I, Beclin1, and SQSTM1/p62 expression were not statistically prominent, suggesting that FoxG1 plays an important role in the regulation of autophagy, which may be necessary for the autophagy activation of N_2_A cells (Fig. 1I–L, *p* < 0.05).

To further verify that FoxG1 activates autophagy, we next measured autophagic flux using the mCherry-EGFP-LC3 adenovirus reporter system in N_2_A cells, in order to detect changes in the numbers of autophagosomes and autolysosomes. Compared to vector transfection group, we found that after pECMV-Foxg1-m-FLAG plasmid transfection in N_2_A cells, the numbers of both autophagosomes and autolysosomes were increased and the latter were more numerous than the former, having the same effect as treated cells with Rap, indicating that autophagy flux was activated by overexpressing FoxG1 in N_2_A cells (Fig. 1M–O, *P* < 0.05).

**Fig. 2:**
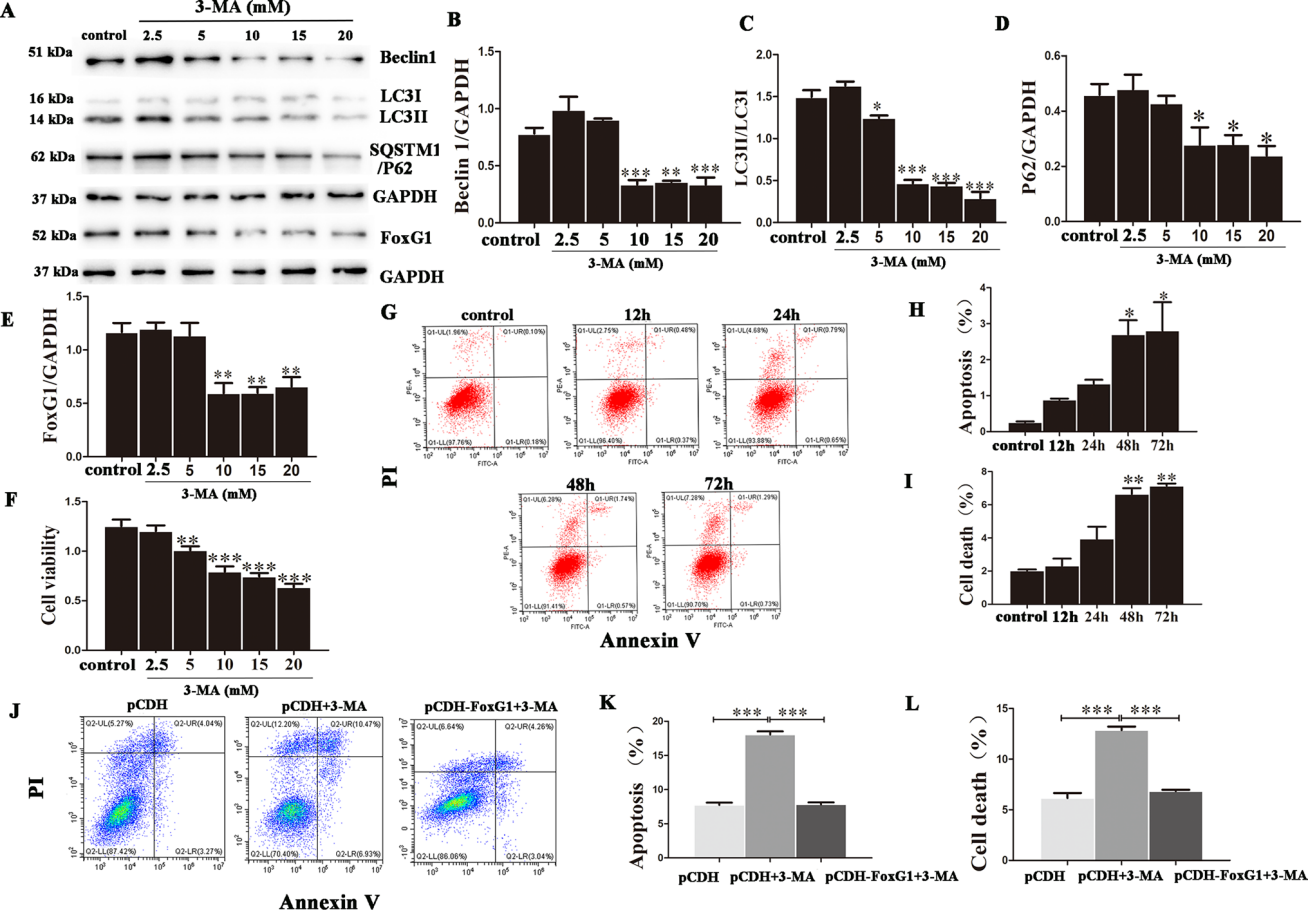
The 3-MA inhibits autophagy and induces cell apoptosis and death and FoxG1 overexpression alleviates these effects. **A** Western blot showing the changes in Beclin1, LC3, SQSTM1/P62, and FoxG1 expression in the N2A cells after different concentrations of 3-MA treatment (2.5 mM, 5 mM, 10 mM, 15 mM, and 20 mM) for 24 h. (**B** [*F*_5,30_ = 25.91, *P* < 0.0001], **D** [*F*_5,30_ = 5.886, *P* = 0.0056], **E** [*F*_5,30_ = 11.90, *P* = 0.0003]). Bar chart indicates the relative expression of these proteins in (**A**). (**C** [*F*_5,30_ = 117.3, *P* < 0.0001]) Bar chart indicates the expression of LC3 II/LC3 I in (**A**). (**F** [*F*_5,30_ = 25.59, *P* < 0.0001]) Cell viability was evaluated by BioTeck. **G** Apoptosis analysis by flow cytometry after 3-MA treatments for different times (12 h, 24 h, 48 h, and 72 h). (**H** [*F*_4,25_ = 7.982, *P* = 0.0213], **I** [*F*_4,25_ = 32.06, *P* = 0.0009]) The proportions of apoptotic cells and dead cells in (**G**). **J** FoxG1/N2A cell lines and pCDH/N2A cell lines were treated with 10 mM 3-MA for 48 h, apoptosis analysis by flow cytometry after treatments. (**K** [F_2,15_ = 251.9, *P* < 0.0001], **L** [F_2,15_ = 104.1, *P* < 0.0001]) The proportions of apoptotic cells and dead cells in J. GAPDH was used as western blot loading control. Values are expressed as means ± SEM. *P < 0.05, **P < 0.01, ***P < 0.001. For each group, *n* = 6/group

We have shown that FoxG1 regulates autophagy activity, and which has been reported to be an effective way to survival of various tissues and cells (He et al. 2019). And having evidences showed that the neuron cells could appear apoptosis and even cell death when the autophagy level was inhibited in several neurodegenerative diseases (Behl et al. 2022). The role of FoxG1 in reducing the cell apoptosis induced by autophagy inhibited has not been cleared. Therefore, we employed autophagy inhibitor 3-MA to explore whether FoxG1 affects cell apoptosis in response to autophagy inhibited. First, we treated the N_2_A cells with different 3-MA concentrations (2.5 mM, 5 mM, 10 mM, 15 mM, 20 mM, 25 mM) for 48 h. Western blot data showed that the level of LC3II, Beclin1, and FoxG1 decreased and SQSTM1/P62 increased when cells treated with more than 10 mM 3-MA (Fig. 2A–E, *P* < 0.05). The CCK8 results also showed that the cell density was obviously decreased compared to the untreated group when the concentration of 3-MA was higher than 10 mM (Fig. 2F, *P* < 0.05). Then, we used propidium iodide (PI) to label the dead cells and Annexin V to label the cells undergoing apoptosis, and the flow cytometry results demonstrated that the proportions of both dead and apoptotic cells were significantly increased compared to the untreated group when the 10 mM 3-MA treatment time was 48 h or longer (Fig. 2G–I, *P* < 0.05). So, we confirmed optimum 3-MA treatment time and concentration for all experiments in this study. Furthermore, we determined the function of FoxG1 in response to 3-MA-induced cell apoptosis and we found that FoxG1 could relieved cell apoptosis and cell death by flow cytometry in FoxG1/N_2_A cells (Fig. 2J–L, *P* < 0.05). Collectively, FoxG1 induces autophagy and alleviates cell apoptosis after autophagy inhibited in N_2_A cells.

### FoxG1 Induces Autophagy via the AMPK/mTOR Pathway in N_2_A Cells

**Fig. 3:**
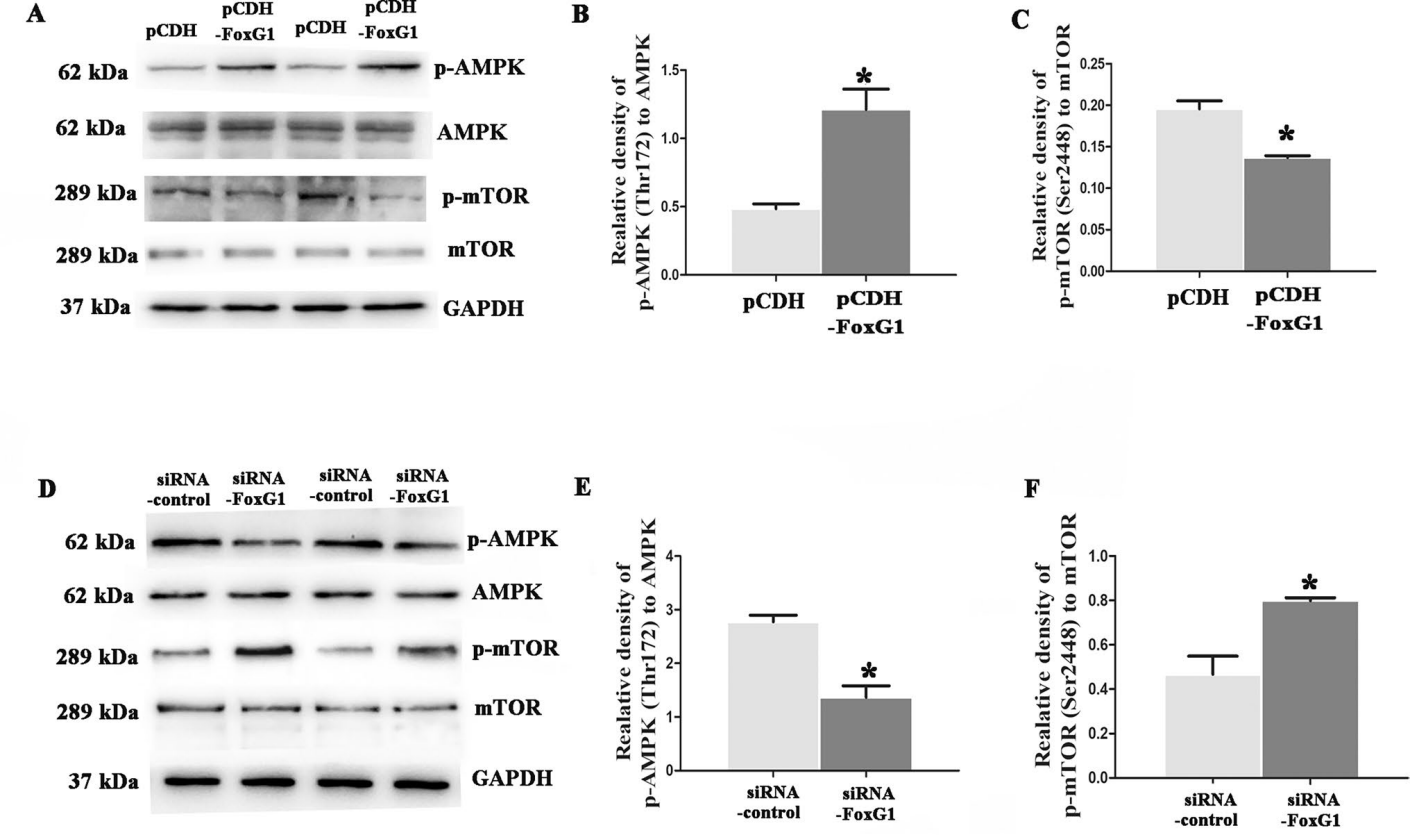
FoxG1 participates AMPK/mTOR pathway. **A** The proteins expression of AMPK, p-AMPK, mTOR, and p-mTOR was checked in FoxG1/N2A cells and pCDH/N2A cells by western blot. (**B** [*t*_10_ = 4.715, *P* = 0.0422], **C** [*t*_10_ = 5.819, *P* = 0.0283]) Bar chart indicates the expression of p-AMPK/AMPK or p-mTOR/mTOR in (**A**). **D** N2A cells transiently transfected with siRNA control or siRNA-FoxG1 for 48 h, and then the cells were harvested for the protein detection of AMPK, p-AMPK, mTOR and p-mTOR. (**E** [*t*_10_ = 5.096, *P* = 0.0364], **F** [*t*_10_ = 5.093, *P* = 0.0365]). Bar chart indicates the expression of p-AMPK/AMPK or p-mTOR/mTOR in D. GAPDH was used as loading control. Values are expressed as means ± SEM. *P < 0.05, **P < 0.01, ***P < 0.001. For each group, *n* = 6/group

AMPK, as the major energy-sensing kinase, activates a large amount of catabolic processes in multicellular organisms, positively regulates autophagy by inhibiting the activity of mammalian target of rapamycin (mTOR). In this study, to investigate whether the upregulation of FoxG1 contributed to the activation of AMPK, we respectively employed FoxG1/N_2_A cells to increase and siRNA-FoxG1 to knock down FoxG1 expression and then detected the expression of p-AMPK and AMPK, and we further explored the effect of FoxG1 on the expression of the key down-stream proteins of AMPK, mTOR. As shown in Fig. 3A–C, Western blot data showed that the level of p-AMPK significantly increased at Thr172 and the level of p-mTOR decreased at Ser2448 in FoxG1/N_2_A cells compared to pCDH/N_2_A cells (*P* < 0.05). As shown in Supplement Fig. 1A–B, Real-time PCR data showed that the level of AMPK significantly increased and the level of mTOR decreased in FoxG1/N_2_A cells compared to pCDH/N_2_A cells(*P* < 0.001). In contrast, when cells treated siRNA-FoxG1 (m-FoxG1-1509) compared to siRNA control, downregulation of FoxG1 expression could inhibit activity of p-AMPK and increase the level of p-mTOR (Fig. 3D–F, *p* < 0.05). Here, we chosen the most efficient one (m-FoxG1-1509) to interfering the expression of FoxG1 (Supplement Fig. 1C–E). Collectively, FoxG1 could activate autophagy via the AMPK/mTOR signaling pathways in N_2_A cells.

### FoxG1 and Autophagy Activity Decrease with NLRP3 Inflammasome Increased as Aβ Peptides Accumulation in N_2_A Cells

**Fig. 4:**
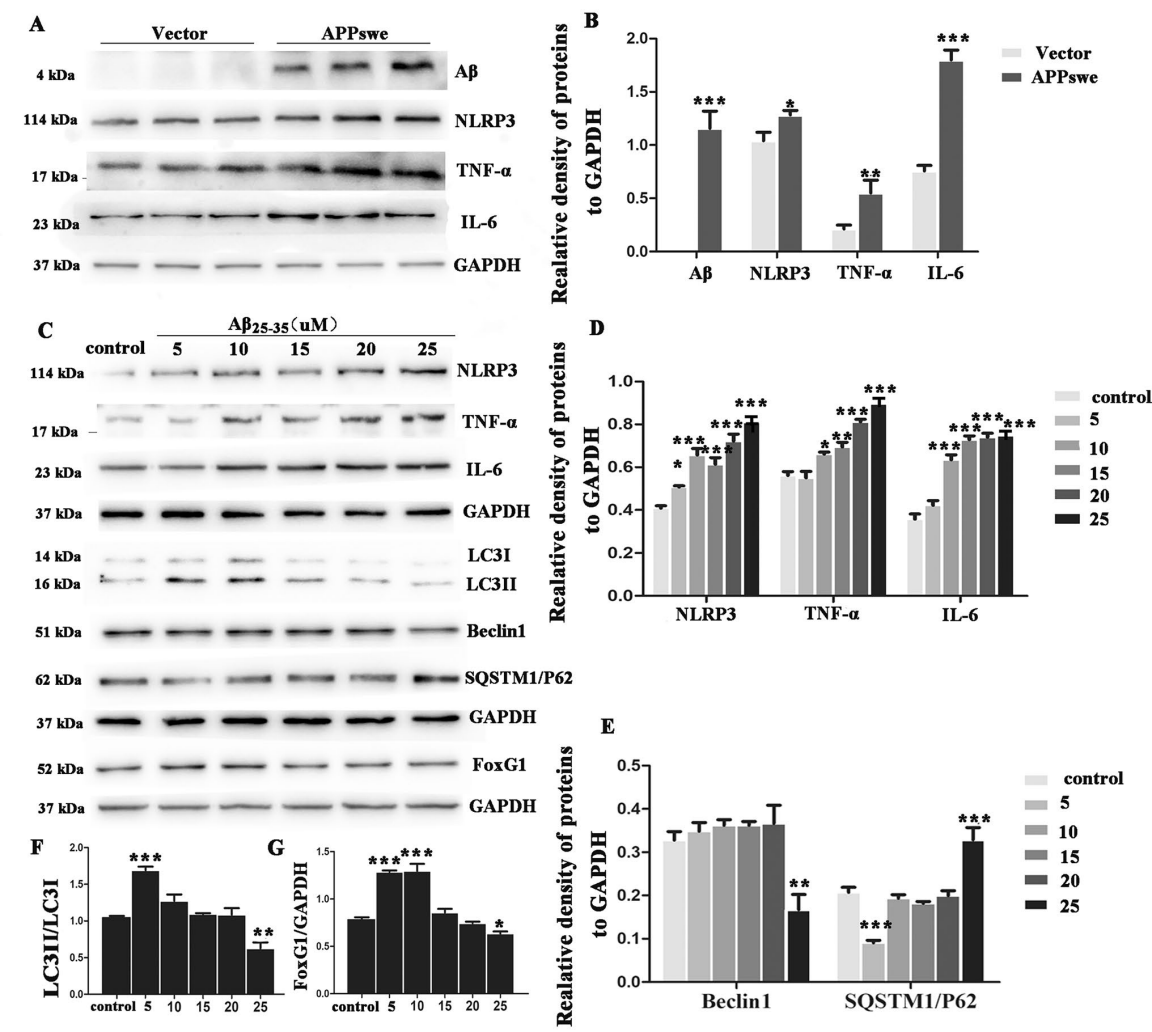
FoxG1 and autophagy activity decrease with NLRP3 inflammasome increased as Aβ peptides accumulation in N_2_A cells. N2A cells transiently transfected with vector or APPswe plasmid for 48 h, cells were harvested to check Aβ protein expression, and then the expression of NLRP3, TNF-α, and IL-6 was studied by western blot. (**B**: NLRP3 [*t*_10_ = 2.790, *P* = 0.0493], TNF-α [*t*_10_ = 4.732, *P* = 0.0091], IL-6 [*t*_10_ = 9.740, *P* = 0.0006]) Bar chart indicates the relative expression of these proteins in (**A**). **C** Western blot showing the changes in NLRP3, TNF-α and IL-6, and Beclin1, LC3, SQSTM1/P62, and FoxG1 expression in the N2A cells after different concentration of Aβ25-35 treatment (5 μM, 10 μM, 15 μM, 20 μM, and 25 μM) for 24 h (**D**: NLRP3 [*F*_5,30_ = 36.55, *P* < 0.0001], TNF-α [*F*_5,30_ = 60.83,* P* < 0.0001], IL-6 [*F*_5,30_ = 64.24,* P* < 0.0001], **E** Beclin1 [*F*_5,30_ = 9.022,* P* = 0.0009], SQSTM1/P62 [*F*_5,30_ = 30.24,* P* < 0.0001], **G** [*F*_5,30_ = 52.89,* P* < 0.0001]) bar chart indicates the relative expression of these proteins in (**C**). (**F** [*F*_5,30_ = 26.53,* P* < 0.0001]). Bar chart indicates the expression of LC3 II/LC3 I in C. GAPDH was used as loading control. Values are expressed as means ± SEM. *P < 0.05, **P < 0.01,***P < 0.001. For each group, *n* = 6/group

Emerging recent evidence indicates that Aβ as a misfolded protein can excite a sustained pro-inflammatory response and further accelerate neuronal death. NLRP3, one of the most common inflammasomes, plays an important role in mediating the neuroinflammation process of neurodegenerative diseases. To test how the level of FoxG1 and autophagy change in response to NLRP3 inflammasome in the cell model of AD, we employed APPswe plasmid transfection to establish AD cellular model in N_2_A cells. We found that transfection of APPswe plasmid could induce Aβ production and the NLRP3 inflammasome has been activated and produced many pro-inflammatory cytokines including IL-6 and TNF-α (Fig. 4A, B, *p* < 0.05). Next, in order to simulate the Aβ accumulation process to a certain extent, we treated the N_2_A cells with different concentrations of 5 μM, 10 μM, 15 μM, 20 μM, and 25 μM Aβ_25–35_ and found the expression of NLRP3, TNF-α, and IL-6 gradually increased with increasing Aβ_25–35_ concentration (Fig. 4C, D, *p* < 0.05). Furthermore, we took advantage of the different concentrations of 5 μM, 10 μM, 15 μM, 20 μM, and 25 μM Aβ_25–35_ to investigate the role of FoxG1. We found that FoxG1 expression as well as autophagy level was first gradually increased with the low-concentration Aβ_25–35_ treatment, but sharply decreased with high-concentration Aβ_25–35_ treatment. As shown in Fig. 4C, E, and F, western blot data showed that the expression of LC3II/ LC3I ratio was increased and P62 was decreased when cells treated with less than 10 μM Aβ_25–35_ and that the expression of LC3II/ LC3I ratio and Beclin1 were decreased and SQSTM1/P62 was significantly increased when cells treated with 25 μM Aβ_25–35_. And the western blot results showed that the expression of FoxG1 was increased when the cells were treated with 5 μM or 10 μM Aβ_25–35_ but was significantly decreased when the cells were treated with 25 μM Aβ_25–35_ (Fig. 4C, G, *p* < 0.05). These results showed that Aβ could induce NLRP3 inflammasome increased and the level of FoxG1 and autophagy decreased, and we speculate that there was a synergistic regulatory relationship between FoxG1 and the autophagy pathway in the Aβ-induced inflammatory response in N_2_A cells.

### FoxG1 Inhibits the Aβ-Induced Activation of NLRP3 Inflammasome via the Upregulation of Autophagy in N2A Cells

**Fig. 5:**
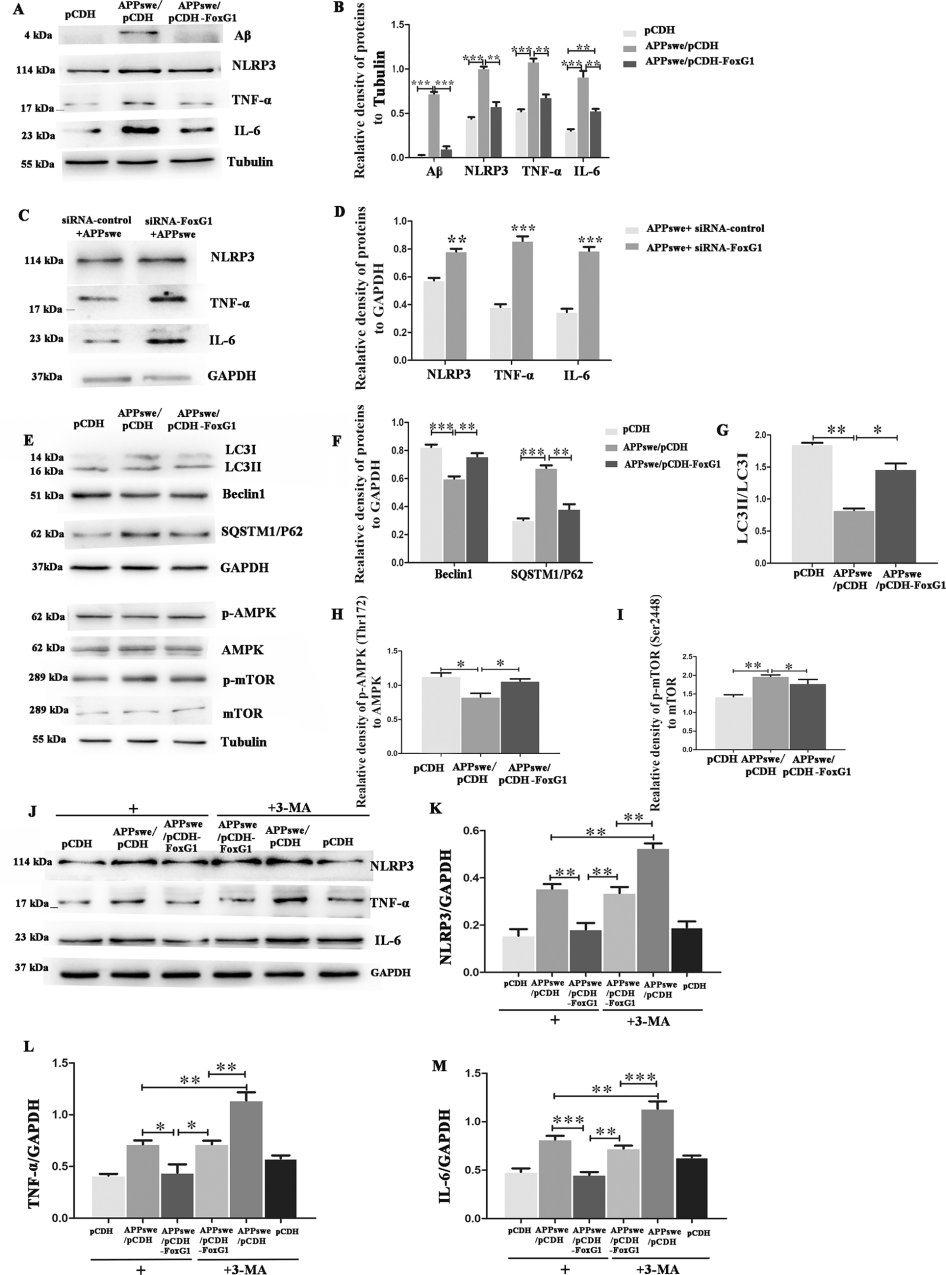
FoxG1 inhibits the Aβ-induced activation of NLRP3 inflammasome via the upregulation of autophagy in N_2_A cells. **A** FoxG1/N2A cells and pCDH/N2A cells are transiently transfected with APPswe plasmid for 48 h. The expressions of Aβ, NLRP3, TNF-α and IL-6 were studied by western blot. (**B**: Aβ [*F*_2,15_ = 367.3, *P* < 0.0001], NLRP3 [*F*_2,15_ = 82.98, *P* < 0.0001], TNF-α [*F*_2,15_ = 79.66,* P* < 0.0001], IL-6 [*F*_2,15_ = 54.51,* P* = 0.0001]). Bar chart indicates the relative expression of these proteins in (**A**). **C** N2A cells transiently co-transfected with siRNA control or siRNA-FoxG1 and APPswe plasmid for 48 h; the expressions of NLRP3, TNF-α, and IL-6 were studied by western blot. (**D**: NLRP3 [*t*_10_ = 7.655,* P* = 0.0016], TNF-α [*t*_10_ = 11.80,* P* = 0.0003], IL-6 [*t*_10_ = 11.58,* P* = 0.0003]). Bar chart indicates the relative expression of these proteins in (**C**). **E** FoxG1/N2A cells and pCDH/N2A cells are transiently transfected with APPswe plasmid for 48 h, the expressions of LC3, Beclin1, SQSTM1/P62 and AMPK, p-AMPK, mTOR, and p-mTOR proteins were studied by western blot. (**F** Beclin1 [*F*_2,15_ = 33.89,* P* = 0.0005], SQSTM1/P62 [*F*_2,15_ = 67.36,* P* < 0.0001]), Bar chart indicates the relative expression of Beclin1 and SQSTM1/P62 in (**E**). (**H** [*F*_2,15_ = 10.19,* P* = 0.0118], **I** [*F*_2,15_ = 13.76,* P* = 0.0057]). Bar chart indicates the expression of p-AMPK/AMPK or p-mTOR/mTOR in (**E**). (**G** [*F*_2,15_ = 8.809, *P* < 0.0076]), Bar chart indicates the expression of LC3 II/LC3 I in (**E**). **J** FoxG1/N2A cells and pCDH/N2A cells transiently transfected with APPswe plasmid were treated with 10 mM 3-MA for 48 h or no 3-MA treatment. After 48 h, cells were then harvested for the protein detections of NLRP3, TNF-α, and IL-6 by western blot. (**K** [*F*_5,30_ = 21.39,* P* < 0.0001], **L** [*F*_5,30_ = 25.39,* P* < 0.0001], **M** [*F*_5,30_ = 34.61,* P* < 0.0001]). Bar chart indicates the relative expression of these proteins in (**J**). GAPDH or Tubulin was used as loading control. Values are expressed as means ± SEM. *P < 0.05, **P < 0.01, ***P < 0.001. For each group, *n* = 6/group

As an important member of the forkhead family of transcription factors, FoxG1 plays an important role in the development, differentiation, and survival of various tissues and cells. But its function in neuroinflammation of AD has not been reported. In this study, by establishing APPswe plasmid transfection-induced N_2_A cellular model, we investigated the inhibition effect of FoxG1 on the activation of NLRP3 inflammasome. We found the level of NLRP3, TNF-a, and IL-6 were downregulated with FoxG1 upregulation in FoxG1/N_2_A cells by western blot compared with in pCDH/N_2_A cells (Fig. 5A, B, *p* < 0.01). In turn, we used siRNA-FoxG1 to further confirm the role of FoxG1 in inflammatory response of AD. The western blot results showed that after APPswe plasmid and siRNA-FoxG1 co-transfection, the level of NLRP3, TNF-a, and IL-6 significantly increased compared with APPswe plasmid and siRNA control transfection group (Fig. 5C, D, *p* < 0.01). These data suggest that the inflammation response was exacerbated by the interference of FoxG1*.* Taken together, FoxG1 exerts an important inhibition effect in Aβ-induced activation of NLRP3 inflammasome in N_2_A cells.

To investigate whether the inhibition effect was associated with the upregulation of autophagy, we employed the autophagy inhibitor 3-MA to decrease the level of autophagy and then examined the level of NLRP3 inflammasome. First, as shown in Fig. 5E, F, Aβ decreased the expression of LC3II/LC3I and Beclin1 and increased the expression of SQSTM1/P62 intensity, which represents autophagy level by transfecting with APPswe plasmid. And we found that overexpression of FoxG1 could relieve these effects (Fig. 5E–G, *p* < 0.05). Aβ inhibited the expression of p-AMPK at Thr172 and activated p-mTOR at Ser2448, and western blot results showed that FoxG1 overexpression also relieved (Fig. 5E, H, I, *p* < 0.05). So, we demonstrated that FoxG1 could activate AMPK pathway and enhance the level of autophagy during APPswe plasmid treatment. Then, we employed 3-MA to inhibit the level of autophagy, and western blot results suggested that the 3-MA could suppress the effect of FoxG1 on the inhibition effect in the protein expression of NLRP3, IL-6, and TNF-a in APPswe plasmid transfection-induced N_2_A cells (Fig. 5J–M, *p* < 0.05). Therefore, these results suggested that FoxG1 inhibited the NLRP3 inflammasome activation via the upregulation of autophagy in Aβ-induced N_2_A cells.

### FoxG1 Regulated the Level of Autophagy in Aβ_25–35_-Induced *Cre/FoxG1*^*fl/fl*^ Mice

**Fig. 6:**
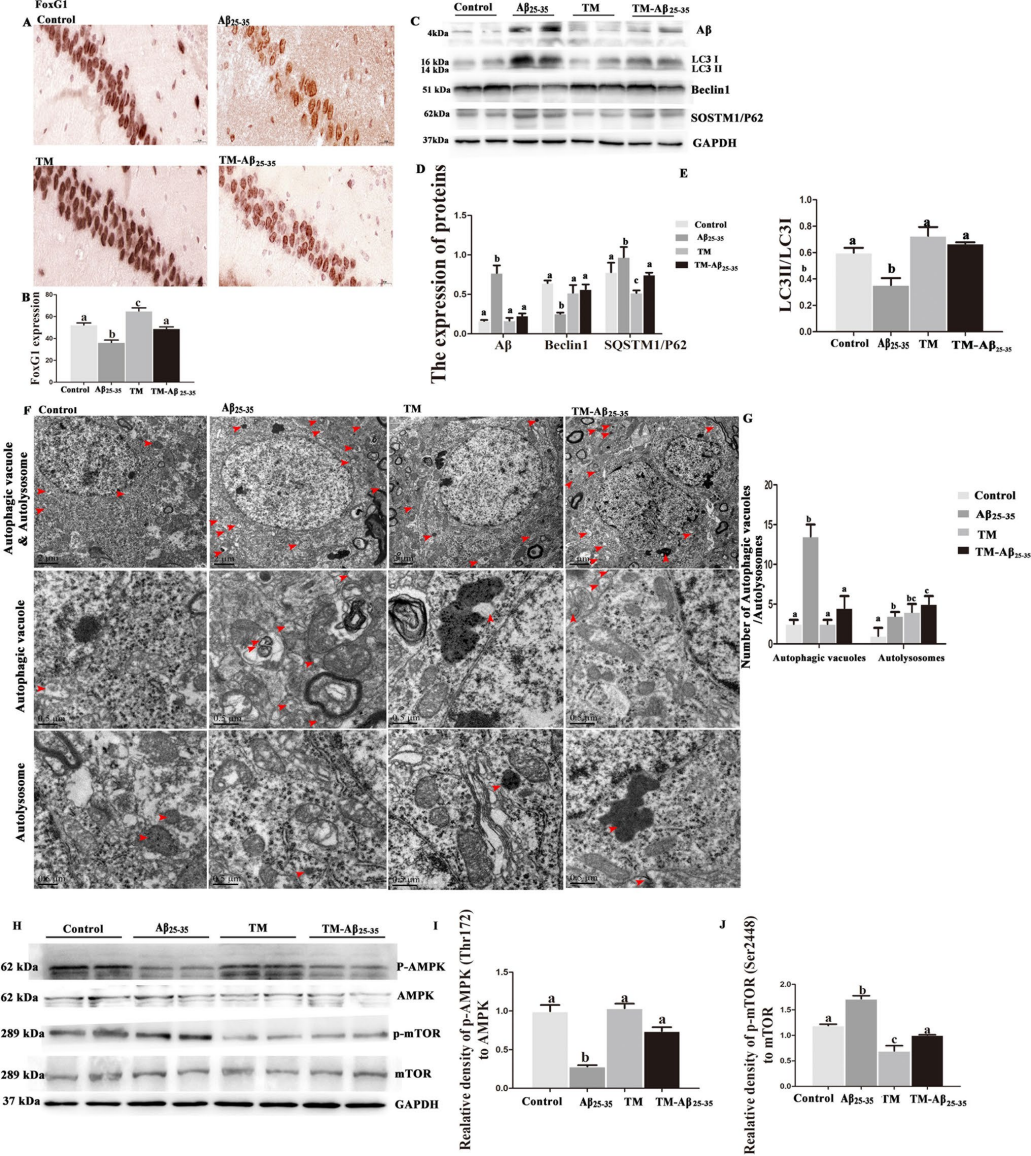
Tamoxifen forced FoxG1 overexpressed in the hippocampus of Aβ_25-35_-induced *Cre/FoxG1*^*fl/fl*^ mice and FoxG1 activated autophagy via AMPK/mTOR pathway. The expressions of FoxG1 in the hippocampal sections was detected by the immunohistochemistry method in four groups. The representative images were captured by a microscope. Scale bar = 20 μm. (**B** [*F*_3,20_ = 29.40,* P* = 0.0001]). Bar chart indicates the qualification of FoxG1 expression. **C** The expressions of Aβ in the hippocampal tissues was detected by the western blot after Aβ25-35 microinjection, and then detected the proteins of LC3, Beclin1 and SQSTM1/P62. (**D**: Aβ [*F*_3,20_ = 29.11,* P* = 0.0001], Beclin1 [*F*_3,20_ = 7.912,* P* = 0.0089], SQSTM1/P62 [*F*_3,20_ = 4.126, *P* = 0.0484]). Bar chart indicates the relative expression of these proteins in (**C**). (**E** [*F*_3,20_ = 12.07, *P* = 0.0179]) Bar chart indicates the expression of LC3 II/LC3 I in (**C**). **F** TEM analysis for evaluating autophagy in four groups. Scale bar = 0.5 μm. (**G** autophagic vacuoles [*F*_3,9_ = 22.07, *P* = 0.0060], autolysosomes[*F*_3,9_ = 3.564, *P* = 0.1256]) Quantification of the autophagic vacuoles and the autolysosomes in (**F**). **H** The expression of AMPK, p-AMPK, mTOR, and p-mTOR proteins were studied by western blot in four groups. (**I** [*F*_3,20_ = 36.91, *P* = 0.0023], **J** [*F*_3,20_ = 48.38, *P* = 0.0013]). Bar chart indicates the expression of p-AMPK/AMPK or p-mTOR/mTOR in (**H**). GAPDH was used as loading control. For all experiments, different letters indicate statistical differences in mean values across groups (*P* < 0.05). Values are expressed as means ± SEM. *P < 0.05, **P < 0.01, ***P < 0.001. For each group, *n* = 6/group

Increasing evidence shows that the level of autophagy is low or even impaired owing to the excessive inflammatory response in the microglia of the brain in AD model mice. To explore the role of FoxG1 in activating autophagy of AD model mice, we extended the in vivo studies by administering i.p. with TM to induce FoxG1 overexpression and simultaneously administering intracerebroventricularly (i.c.v.) with Aβ_25–35_ peptides in *Cre/FoxG1*^*fl/fl*^ mice to induce AD *vivo* model. Firstly, we randomly divide the *Cre/FoxG1*^*fl/fl*^ mice into four groups (7 mice in each group): the sham-operated control group, Aβ_25–35_-intoxicated group (Aβ_25–35_ group), TM-treated group (TM group), TM and Aβ_25–35_-intoxicated group (TM-Aβ_25–35_ group). Immunohistochemistry result showed that TM successfully induced FoxG1 overexpression by using anti-FoxG1 antibody (Fig. 6A, B, *p* < 0.05). First, Aβ_25–35_ successfully induced Aβ production, and then we checked the autophagy-related proteins including LC3, Beclin1, and SQSTM1/P62 by western blot, found that TM-Aβ_25–35_ group significantly showed increased the expression of LC3II/LC3I ratio and Beclin1, and decreased the protein SQSTM1/P62 and Aβ compared with Aβ_25–35_ group (Fig. 6C–E, *p* < 0.05). To double confirm that FoxG1 regulates the autophagy pathway as part of the Aβ_25–35_-induced inflammatory response, we used TEM imaging to show the changes in autophagy and after overexpression of FoxG1. The imaging showed that the hippocampus area of Aβ_25–35_ group mice had more autophagic vacuoles and autolysosomes compared with control group (Fig. 6F, G), overexpression of FoxG1 decreased the number of autophagic vacuoles, but increased the number of autolysosomes compared with Aβ_25–35_ group mice (Fig. 6F, G, *p* < 0.05), indicating that autophagic flux is activated by FoxG1. Furthermore, we made a thorough inquiry in the activation of p-AMPK and p-mTOR in four groups. Western blot results showed that the protein of p-AMPK was decreased and p-mTOR was increased in Aβ_25–35_ group and FoxG1 overexpression could alleviate the change of Aβ_25–35_-induced (Fig. 6H–J, *p* < 0.05). Taken together, FoxG1 overexpression could activate autophagy in Aβ_25–35_-induced mice of AD *vivo* model.

### FoxG1 Improves the Cognitive Function and Inhibits the NLRP3 inflammasome in Aβ_25–35_-Induced *Vivo* Model

**Fig. 7:**
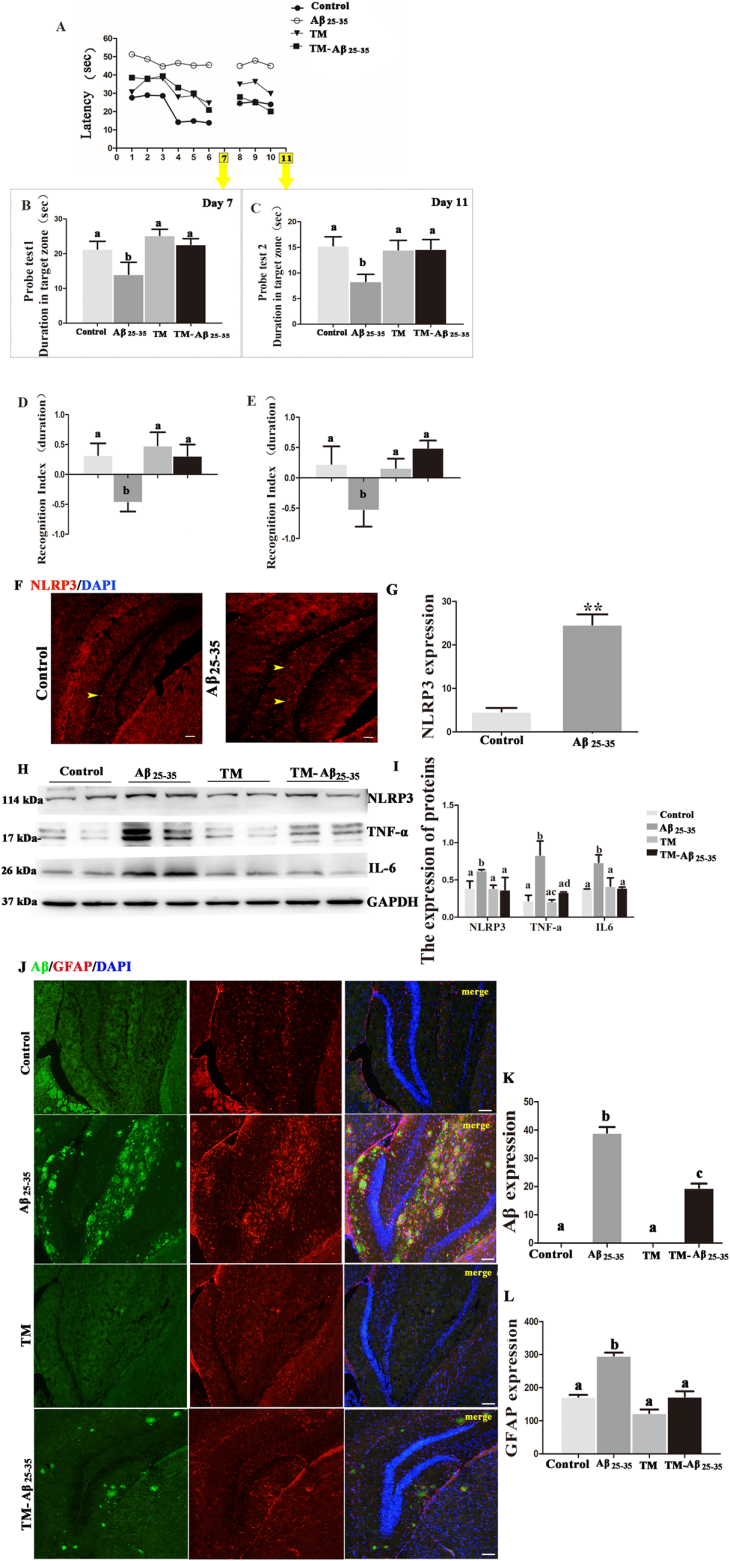
FoxG1 improves the cognitive function and inhibits the NLRP3 inflammasome in Aβ_25-35_-induced Cre/FoxG1^fl/fl^ mice. Representative images of escape latency (**A**) and time spent in the target platform quadrant in probe 1 (**B** [*F*_3,20_ = 3.732, *P* = 0.0247]) and in probe 2 (**C** [*F*_3,20_ = 3.573, *P* = 0.0288]) were recorded and obtained by the Morris water maze (MWM) test system. Bar charts indicate the recognition index in novel object recognition (**D** [*F*_3,20_ = 5.178, *P* = 0.0109]) and in novel place recognition (**E** [*F*_3,20_ = 4.094, *P* = 0.0247]). Data are presented as mean ± SEM, *n* = 6/group. After behavioral experiments, the mice were anesthetized and taken brain tissue. **F** Immunofluorescence staining with NLRP3 antibody in the hippocampus from four-group mice. (**G** [*t*_10_ = 8.018, *P* = 0.0013]) Quantification of the NLRP3 spot number in (**F**). **H** The expression of NLRP3, TNF-α, and IL-6 were studied by western blot. GAPDH was used as loading control. (**I**: NLRP3 [*F*_3,20_ = 1.593, *P* = 0.3238], TNF-α [*F*_3,20_ = 8.426, *P* = 0.0334], IL-6 [*F*_3,20_ = 5.217, *P* = 0.0722]). Bar chart indicates the relative expression of these proteins in (**H**). **J** Immunofluorescence co-staining with Aβ antibody and GFAP antibody in the hippocampus from four-group mice, GFAP spots were emerged with Aβ depositions. Quantification of the (**K** [*F*_3,20_ = 223.1, *P* < 0.0001]) Aβ plaque number and (**L** [*F*_3,20_ = 44.01, *P* = 0.0016]). GFAP spot number in (**J**). For all experiments, different letters indicate statistical differences in mean values across groups (*P* < 0.05). Values are expressed as means ± SEM. *P < 0.05, **P < 0.01, ***P < 0.001. For each group, *n* = 6/group

Cognitive decline is the main symptom of AD mice. In addition, emerging evidences shows that NLRP3 inflammasome is activated in the microglia of AD model mice. To validate Aβ_25–35_-induced cognitive impairment and the effect of FoxG1 in *vivo* model, we conducted the MWM test, novel object, and novel place learning tests to evaluate the learning and memory functions of the mice (Fig. 7). As shown in Fig. 7A, escape latency in Aβ_25–35_ group mice was significantly longer than that in control group (*P* < 0.05), and we found that FoxG1 overexpression could reduce escape latency in TM-Aβ_25–35_ group compared with Aβ_25–35_ group (*P* < 0.01). At the same time, in probe test 1 and probe test 2, Aβ_25–35_ group mice both showed less time to cross in the platform quadrant than control group mice, while FoxG1 overexpression mice also increased the time spent in crossing in the platform quadrant (Fig. 7B, C, *p* < 0.05). Then, we used novel object and novel place learning tests to explore spatial memory functions of the mice. In Fig. 7D, Aβ_25–35_ group mice showed a remarkable reduction in duration of novel object than control group mice, but FoxG1 overexpression mice showed a significant increase in recognition index compared to Aβ_25–35_ group mice (*P* < 0.05). Again, Aβ_25–35_ group mice displayed a significant deficit in novel place recognition, as shown by lower preference for a novel place compared to control group mice, and the same FoxG1 overexpression could be improved in Fig. 7E (*P* < 0.01). Taken together, these data indicated that FoxG1 could improve the cognitive function of Aβ_25–35_-induced mice model.

According to the results above, we have suggested that FoxG1 increased autophagy level in Aβ_25–35_-induced mice. In addition, in this study, we employed Aβ_25–35_-induced mice to further confirm the effect of FoxG1 on the inhibition of NLRP3 inflammasome in *vivo*. We used western blot and immunofluorescence to detect the level of NLRP3 inflammasome and other inflammation-associated proteins including IL-6, TNF-α, and GFAP in hippocampus brain area of the mice. In Fig. 7F–I, Aβ_25–35_ induced a high level of NLRP3 expression by immunofluorescence and western blot compared with control group (*P* < 0.05). In TM-Aβ_25–35_ group, FoxG1 overexpression decreased the expression of NLRP3, meanwhile downregulated the expression of IL-6 and TNF-α by western blot compared with Aβ_25–35_ group (Fig. 7F–I, *p* < 0.05). Then immunofluorescence results showed that GFAP appeared surrounding Aβ deposition, FoxG1 overexpression could reduce GFAP expression, and reduce Aβ plaque compared with Aβ_25–35_ group (Fig. 7J–L). In brief, FoxG1 could have anti-inflammation effect in Aβ_25–35_-induced *vivo* model. Collectively, FoxG1 could improve the cognitive function and inhibit the activation of NLRP3 inflammasome and reduce Aβ plaque deposition via the activation of autophagy in Aβ_25–35_-induced mice model.

## Discussion

Alzheimer’s disease, being the primary type and underlying factors of dementia, is responsible for absolute majority of all cases of dementias(Wu et al. 2020). AD is a neurodegenerative condition marked by gradual loss of memory and decline in cognitive abilities(Friedland and Chapman 2017). The deposition of Aβ and hyperphosphorylation of Tau are the critical pathological features of AD (Martínez et al. 2012). Stacking of these misshapen pieces in brain cells leads to the death of neurons and speeds up the advancement of neurodegeneration. The Aβ peptide is a substance that creates insoluble clumps which have a tendency to accumulate on the cell surface (Abedin et al. 2021). Pathological deposition is caused by non-physiological cleavage of APP by BACE1 (β-site amyloid precursor protein cleaving enzyme-1; also known as β-secretase). Research has indicated that neurotoxic peptides originating from (APP), specifically Aβ1–42, play significant roles in AD (Imai et al. 2021). Mice are intracerebroventricularly (i.c.v.) injected with Aβ1–42 or Aβ25–35, highly neurotoxic components derived from (APP), extensively utilized in fundamental studies to the prevent and manage AD (Schmid et al. 2017). Additionally, research demonstrated that improperly folded proteins can also trigger a persistent pro-inflammatory reaction and further hasten the death of neurons (Thellung et al. 2018). For this research, we employed mice injected with Aβ_25–35_ peptide (i.c.v.) to mimic the inflammatory reaction and cognitive dysfunction in a mouse model, and we also administered the Aβ25–35 peptide at N2A to induced cell models. The inflammation caused by NLRP3 inflammasome extensively reported and exhibited to be strongly linked to the deterioration and advancement of AD (Feng et al. 2020). Furthermore, the NLRP3 inflammasome activation in microglia can enhance the formation of the Aβ-ASC complex by facilitating the binding of the adapter protein ASC with Aβ (Venegas et al. 2017). This, in turn, exacerbates the aggregation of Aβ and accelerates to the progression of AD. As a result, it has been crucial to focus on suppressing neuroinflammation mediated by the NLRP3 inflammasome as a key approach in preventing and treating AD.

The process of autophagy involves engulfment and degradation of devastated macromolecules and organelles, including misfolded proteins and useless mitochondria by lysosomes. This process helps maintain intracellular environmental homeostasis and promotes cell survival (Jacobs et al. 2020; Qiu et al. 2020). The interaction between autophagy and inflammation is primarily discussed at the inflammatory disease, especially in IBD (Larabi et al. 2020) and type 2 diabetes (Korbut et al. 2020). While autophagy and inflammation may be both involved in central nervous system. Recent findings indicate that in astrocytes and microglia, the activation of inflammatory factors triggers autophagy, resulting in the buildup of numerous acidic vesicles containing autophagic markers (p62 and LC3) specifically in microglia(Ito et al. 2021).Autophagy additionally plays a crucial part in inhibiting neuroinflammation (Wang et al. 2020b). The autophagosome engulfs and degrades the NLRP3 inflammasome which functions as a substrate for autophagy (Ahmed et al. 2017). According to reports, the activation of autophagy in microglia or neurons effectively inhibits the NLRP3 inflammasome associated with AD pathology. In addition, activation of autophagy flux also enhances the removal of Aβ (Saha et al. 2021). So, autophagy activation could be used to downregulate the NLRP3 inflammasome and further alleviate the pathology of AD. Meanwhile, reports found that autophagy inducers, such as metformin (Wang et al. 2020c), were reported that the NLRP3 inflammasome was inhibited by the activation of autophagy.During this investigation, we utilized the autophagy stimulant Rapamycin to enhance the autophagy level, resulting in an elevation of Beclin1 expression and an increased ratio of LC3II/LC3I in N2A cells. In the contrary, we choose autophagy inhibitor 3-methyladenine to downregulate autophagy level and demonstrated low levels of autophagy could induce apoptosis and death in N2A cells. Hence, the quest for a potent autophagy stimulant is considered a beneficial approach to combat AD through the constraint of NLRP3 inflammasome.

The FOXG1 gene, belonging to the forkhead family, plays a crucial role as a transcription factor in controlling the growth and specialization of cells (Hettige et al. 2022). Mutations in FOXG1 gene have a negative impact on the development and differentiation of axons and neurons, including microcephaly, seizures, and profound intellectual disability. On the other hand, glioblastoma often shows an elevated expression of FoxG1 (Richard and Jia-Hao 2020). Previous research has shown that FoxG1 regulates the cell cycle in AD, including (1) by preventing cells from reentering the cell cycle (G0 → G1) through the inhibition of p21-activated kinase (PAK3), and (2) reducing the expression of cell cycle markers, thereby controlling cell cycle progression (Wang et al. 2022b). Additional research has discovered that FoxG1 plays a crucial part in governing the survival of nerve cells (Miyoshi et al. 2021). The level of FoxG1 is closely relevant to mitochondrial autophagy proteins, indicating its role in the functional regulation of mitochondria which linked to the process of aging and inflammation (He et al. 2021). However, there have been no reports on whether it activates autophagy to inhibit inflammation of AD. Here, we speculated that FoxG1 might also be involved in anti-inflammatory and pro-survival processes through autophagy pathway during Aβ25–35-induced neuroinflammation. In vitro studies, we firstly constructed the cell line stably expressing FoxG1 in N2A cells (named as FoxG1/N2A cells) to explore whether FoxG1 activates autophagy; we found that the changes of Beclin1 and LC3 and SQSTM1/P62 proteins in FoxG1/N2A cells were consistent with the rapamycin treatment, rapamycin acts as a positive control for autophagy activation. Subsequently, it was discovered that exposing N2A cells to minimal doses of Aβ25–35 for brief durations can elicit an inflammatory reaction; however, it triggered the activation of FoxG1 expression. As the concentration or duration of Aβ25–35 increased, there was a significant decrease in the expression of FoxG1 and a significant increase in apoptosis levels in N2A cells. Further investigation and verification of this relativity are proved in mouse models in vivo.

Furthermore, apart from the suppression of the inflammatory reaction through the activation of autophagy in N2A, numerous studies have indicated that FoxG1 can regulate autophagy in various cell types, particularly in inner hair ear cells and glioma cells. Inhibition of FoxG1 leads to a decrease in the production of LC3II in the hair cells undergoing mimetic aging in response to lipopolysaccharide (LPS) treatment (He et al. 2021). According to research, FoxG1 vigorously regulates in preventing radiation-induced cell death in malignant glioma cells by controlling autophagy. This suggests that FoxG1 could be a potential target for treating brain glioma in humans (Xiao et al. 2021). AMPK, which serves as a key controller of cellular energy balance, is frequently documented to promote autophagy by directly suppressing mTOR function or through alternative pathways (Abad-Jiménez et al. 2022). During this investigation, it was discovered that the increase in FoxG1 expression triggers the activation of the AMPK signaling pathway, subsequently leading to the suppression of mTOR signaling. Knocking down FoxG1 significantly reduced the p-AMPK expression at Thr172 while increasing p-mTOR expression at Ser2448, consequently suppressing AMPK-mediated autophagy. In the meantime, several researchers have discovered that compound C, which is an inhibitor of AMPK, also boosts the conversion of LC3II (Aizawa et al. 2017). Therefore, FoxG1 may induce autophagy via the activation of AMPK/mTOR signaling pathway. The primary objective of the present research was to examine whether FoxG1 has the ability to suppress neuroinflammation induced by Aβ25–35 and elucidate its mode of operation. In this study, FoxG1 demonstrated a strong suppressive impact on the activation of NLRP3 inflammasome in N2A cells induced by Aβ_25–35_, consequently preventing the subsequent cell death of N2A cells. At the same time, we utilized AD model mice (aged 6 months old) induced by Aβ25–35, which displayed notable cognitive impairments, to examine the neuroprotective impact of FoxG1. The findings indicated that FoxG1 had the ability to reduce the time spent by mice to escape and increase the duration they spent in the platform quadrant during MWM test. Additionally, it enhanced the recognition index in both the novel object and novel place learning tests for the Aβ25–35-induced AD mice. Moreover, it was discovered that FoxG1 suppressed the activation of NLRP3 inflammasome, as well as TNF-α and IL-6, potentially through the induction of autophagy. Hence, these indications suggest that FoxG1 has the potential to enhance the cognitive abilities and reduce neuroinflammation in mice with AD.

## Conclusion

To sum up, this research validated the neuroprotective impact of FoxG1 in AD and elucidated that FoxG1 suppressed the activation of NLRP3 inflammasome induced by Aβ_25–35_ via activating autophagy through AMPK/mTOR pathway. Hence, the activation of FoxG1 is proved to be a valuable approach that has the potential to be further enhanced as a new contender in the treatment of AD.

## Supplementary Information

Below is the link to the electronic supplementary material.

Supplementary Material Bar charts indicate the expression of AMPK mRNA (A [t_10_ = 10.64, P = 0.0004]) and mTOR mRNA (B [t_10_ = 10.69, P = 0.0004]) in pCDH/N2A cells and FoxG1/N2A cells. Choosing the most efficient one to interfering the expression of FoxG1 among siRNA-FoxG1 1-3 (m-FoxG1-1141, m-FoxG1-1509 and m-FoxG1-1990). (C [F_3,20_ = 12.01, P = 0.0025]) Real-Time PCR data showed FoxG1 expression was decreased by 77.4% and 59.3% after respectively transfecting m-FoxG1-1509 and m-FoxG1-1990 compared with siRNA-control in transcriptional level. (D, E [F_3,20_ = 3.380, P = 0.1350]) Western blot data showed FoxG1 protein expression was decreased 46% after transfecting m-FoxG1-1509 compared with siRNA-control. GAPDH was used as loading control. So, the m-FoxG1-1509 was selected as the most efficient interfering RNA for knocking down FoxG1 in this paper. Values are expressed as means ± S. E. M. *p < 0.05, **p< 0.01,***p< 0.001. For each group, n = 6/group (TIF 965 KB)

## Data Availability

In this study, all experiments have been repeated and all data are authentic and reliable. Data availability can be contacted with yunqiwork@outlook.com.
