# Supplementary figures and images for: FoxG1 as a Potential Therapeutic Target for Alzheimer’s Disease: Modulating NLRP3 Inflammasome via AMPK/mTOR Autophagy Pathway
Source: Cell Mol Neurobiol. 2024 Apr 17;44:35. doi: 10.1007/s10571-024-01467-4 (PMC11023968; doi:10.1007/s10571-024-01467-4)

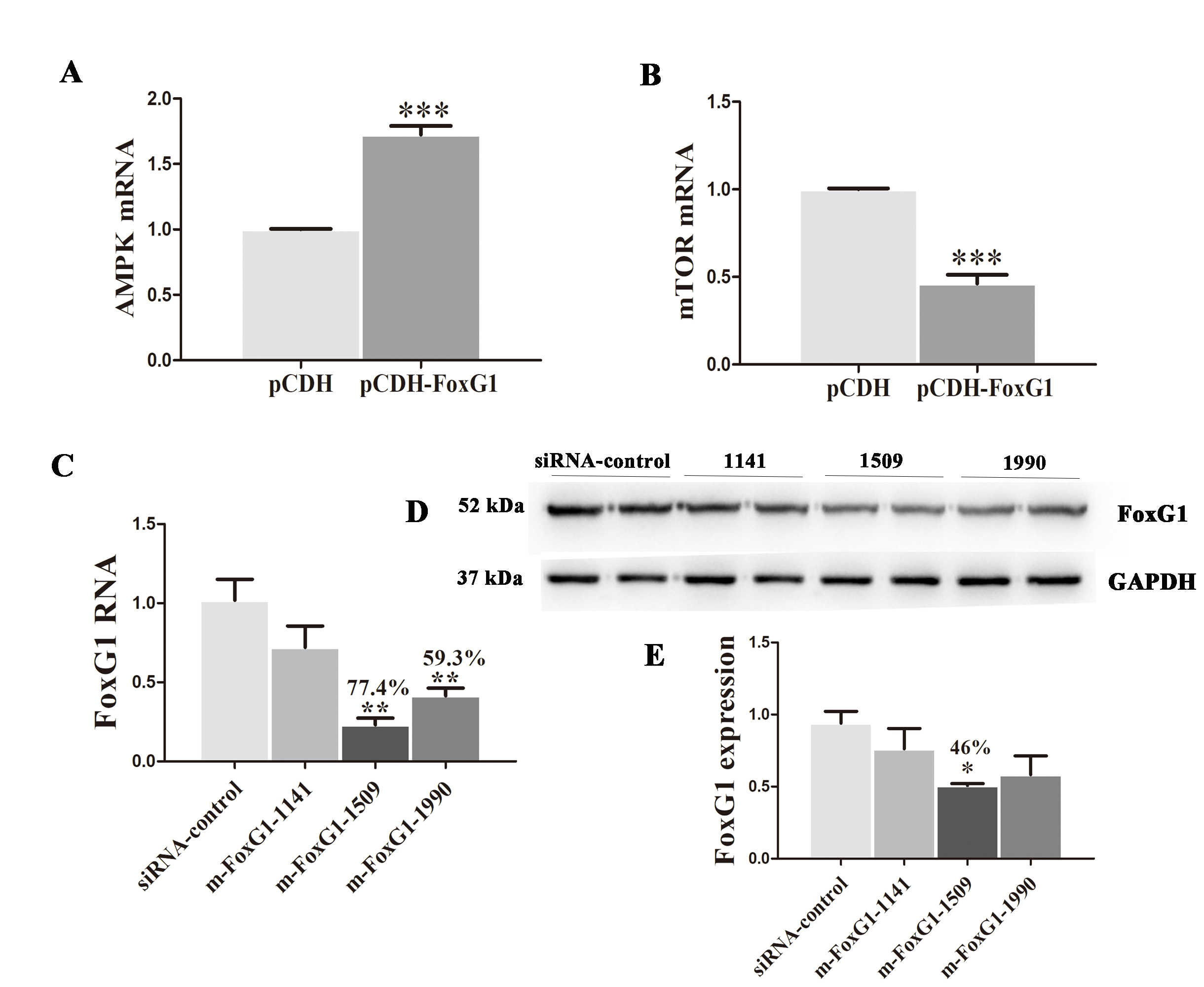

Supplement: Supplementary file 1 — Supplementary Material Bar charts indicate the expression of AMPK mRNA (A [t10 = 10.64, P = 0.0004]) and mTOR mRNA (B [t10 = 10.69, P = 0.0004]) in pCDH/N2A cells and FoxG1/N2A cells. Choosing the most efficient one to interfering the expression of FoxG1 among siRNA-FoxG1 1-3 (m-FoxG1-1141, m-FoxG1-1509 and m-FoxG1-1990). (C [F3,20 = 12.01, P = 0.0025]) Real-Time PCR data showed FoxG1 expression was decreased by 77.4% and 59.3% after respectively transfecting m-FoxG1-1509 and m-FoxG1-1990 compared with siRNA-control in transcriptional level. (D, E [F3,20 = 3.380, P = 0.1350]) Western blot data showed FoxG1 protein expression was decreased 46% after transfecting m-FoxG1-1509 compared with siRNA-control. GAPDH was used as loading control. So, the m-FoxG1-1509 was selected as the most efficient interfering RNA for knocking down FoxG1 in this paper. Values are expressed as means ± S. E. M. *p < 0.05, **p< 0.01,***p< 0.001. For each group, n = 6/group (TIF 965 KB) [file 10571_2024_1467_MOESM1_ESM.tif]
